# Annotations

**Published:** 1892-07-23

**Authors:** 


### Annotations.

**Flowers, and Flower-Girls.**

"The trade in cut flowers in London alone is of the amount of £5,000 a day." This statement, startling as it seems, is taken from a sufficiently reliable source: the report of the Flower-Girls' Mission. Allowing for the large proportion of this sum with which the florists' shops may be accredited, there remains a very considerable sum to represent the investment and earnings of the flower-girls who besiege our steps at every corner. "Flower-girls" we call them, though for many girlhood is a thing of the past—hard-working mothers of families, whose children's food depends on a few cheap leaves and blossoms. It is only when flowers are cheap that the street flower seller dare buy. Early in the morning, between four and six a.m., she is at Covent Garden, waiting till the buyers from the shops have made their selection, to price and cheapen what remains. In winter, when flowers are expensive, all that she dare venture on are often fallen blooms and broken scraps of fern, which she will nevertheless twist with skilful fingers, aided by wire, into "button-holes" that are dainty enough to look at. She has never, at the best, much capital to invest, and her wares are liable to destruction alike from winter's frost and summer's glare. The outside world that only buys the roses and violets, little knows how hard a struggle these girls have to keep hunger, the wolf, and temptation, the serpent, at bay; and the best that the outside world can do is to be thankful—practically thankful, like the Quaker who was "thanful five dollars"—that there exists a Flower Girls' Mission, with the Earl of Aberdeen for president, which has its head-quarters in a room in Lockhart's Cocoa Rooms in Covent Garden. There the girls are warmed and fed, while they receive such help and counsel as may enable them to wear "the white flower of a blameless life" throughout their laborious days. Some are received into a home, where they are taught artificial flower-making; a trade at which a girl may earn from twelve to twenty-five shillings a week, but which is too dependent on the caprices of fashion. As many as possible are drafted into domestic service, the safest and most natural occupation for a young woman to follow. We cannot here detail all that is done by the mission, but we commend it to those who love "the flowers so blue and golden, stars which in earth's firmament do shine," and are therefore interested in those who bring them into city streets and city homes.

**Election Fever.**

It is passing away, but there is no doubt that the epidemic has been a severe one—as severe as the influenza, and and infinitely more contagious. We mean that fever of excitement which burst out at the Dissolution, was marked by great eruptions of oratory and of blue and red ribbons, and by the severing of acquaintance between people who had jogged along pleasantly for seven years past, but who are now convinced that their neighbours of the opposite political faction are fit only to be incarcerated in a lunatic asylum—and, for preference, Broadmoor. The electorate has been at a white heat, the non-electorate not much cooler. The butcher's boy and the youth who drives the grocer's van settled their political differences on the spot in the good old British fashion. In the kitchen, cook and housemaid grow warm over the merits of the factions they severally support, and since they have not votes themselves, they use all their influence—of the kind made immortal by the fair Duchess of Devonshire a hundred years ago—to influence their sweethearts to vote as they desire them. Business men, usually as regular as the clock in the performance of their duties, are to be seen in the middle of the working day miles away from office, shop, or warehouse, all ordinary duties neglected for the time for the sake of attending meetings, urging apathetic voters to go to the poll, or waiting at clubs or newspaper offices to hear the latest results. Here and there we may meet with cases which justify the wail of that good woman who wrote to Mr. Balfour that she hoped he would prevent the Dissolution, because, when it came, it would make her husband dissolute: but, on the whole, there is nothing in a General Election to make a Briton ashamed of his country and her modes of government. Bitterness there has been and, perhaps, calumny, as happens in heated debate on less important topics; but there has been something that more than atones for the hasty speaking of candidates and their supporters. There has been an enlarging of sympathy; a consciousness of something more than individualism, more even than parochialism in our being; a realization of the fact that we are one nation, that each cotter, miner, villager, has a share in the national life, and throbs with the pulsations of a kingdom. To quicken the sense of national life, to develop the sense of national responsibility, to bring out the feeling of a wider unity through all the conflict of individual interests; these are gains not counted at the poll, but they are results worth achieving at the cost of even the stress and strain of an attack of election fever.

**A Lifeboat Saturday.**

A plea has gone forth for a Lifeboat Saturday and Sunday. The need is great, and the claim is justified, whilst in no sense can it be regarded as a rival to the hospital movement. The two services walk hand in hand. The instruments and the field of action are different, but the goal of both—the saving of human life—are the same. Indeed, often does the hospital continue what the lifeboat has begun. In the need for assistance from the length and breadth of the land they are one also. In the benefits which the Hospital Saturday and Sunday Funds have conferred on the hospitals, the lifeboat service would have a share also. The life and interest which these movements have infused in our whole hospital system is also essential to the lifeboat service if it is to continue and increase its record of the past. No class of men call forth our more ardent sympathies and our admiration than those who devote their existence to saving lives from shipwreck, but we, few of us, participate as we might in a noble work by opening our purses to help it on. As the years go round and the work of rescue is carried steadily on, unostentatiously, but increasingly and effectually, the record shows two remarkable facts—one, that the little town of St. Anne's-on-Sea, with its population of only 2,500, raises more money than any important inland town in the kingdom; and, secondly, when a great disaster occurred to a lifeboat crew, and public attention was especially called to the wants of the families of those who thus lost their lives for others, £30,000 was subscribed in a surprisingly short time. These two facts point to a case of "out of sight, out of mind." Our sympathies can be awakened as warmly as are those of the dwellers of the little town by the sea. The claims of the lifeboat men on us who live inland are just as great. But we want these claims brought before us to be told what is being done, what has been done. Facts are stronger than imagination, and these, when a yearly collection is made on behalf of the lifeboat service, could be brought before us in our churches. Since the foundation of the Institution in 1824, 36,000 lives have been saved. Last year over 500 rescues were effected. All will, we feel sure, unite with Mr. Macara, who has already so successfully started Lifeboat Saturday in Manchester, Liverpool, Oldham, Stockport, and Bolton, in furthering a wider scheme throughout the kingdom, and the amount of fresh interest in a greater knowledge of so noble a work, together with a sense of participation, will more than compensate for our lightened purses.

